# Prehospital reversal of profound respiratory acidosis and hypercapnic coma by non-invasive ventilation: a report of two cases

**DOI:** 10.1186/s12245-020-00284-y

**Published:** 2020-05-07

**Authors:** PE Fubini, L Suppan

**Affiliations:** Division of Emergency Medicine, Department of Anaesthesiology, Clinical Pharmacology, Intensive Care and Emergency Medicine, Geneva University Hospitals and Faculty of Medicine University of Geneva, CH-1211, Geneva, Switzerland

**Keywords:** Acute respiratory failure, Altered mental status, Non-invasive ventilation, Respiratory acidosis

## Abstract

**Background:**

In chronic obstructive pulmonary disease (COPD) patients with acute respiratory failure (ARF), non-invasive ventilation (NIV) is generally recommended and has proven its benefits by reducing endotracheal intubation (ETI) rates, intensive care unit (ICU) admissions, complications, and mortality. Choosing between immediate ETI or NIV trial is often difficult when such patients present with an altered mental status. Some guidelines recommend avoiding NIV when consciousness is impaired given the risk of aspiration, and some authors suggest that a pH < 7.25 is highly predictive of NIV failure. Though clinical response to a well-adjusted NIV treatment can be both swift and spectacular, these contraindications probably encourage physicians to proceed to immediate ETI. Some studies indeed report that NIV was not even considered in as many as 60% of patients who might have benefited from this therapy, though ETI related complications might have been avoided had NIV been successfully applied.

**Case presentation:**

We report two cases of ARF in COPD patients who were successfully treated by NIV in prehospital setting and avoided ETI despite contraindications (altered mental status with a Glasgow Coma Scale < 8) and failure risk factors (severe respiratory acidosis with pH < 7.25).

**Conclusion:**

In COPD patients presenting ARF, NIV trial could be considered even when relative contraindications such as an altered level of consciousness or a severe respiratory acidosis are present.

## Background

Acute respiratory failure (ARF) in chronic obstructive pulmonary disease (COPD) patients is a well-known indication for non-invasive ventilation (NIV) trial [1]. The benefits provided by this therapy are numerous and well-studied, the most prominent being reductions in mortality as well as in endotracheal intubation (ETI) and intensive care unit (ICU) admission rates [2, 3]. The main contraindication to NIV is the need for emergent ETI, considered to be mandatory when consciousness is severely impaired, when the patient is unable to cooperate, or when the airways need to be protected because of a high aspiration risk [4]. Unfortunately, COPD patients with ARF often meet these criteria because of hypercapnic coma, and the role of NIV in this setting is therefore still debated [5, 6]. Furthermore, some authors suggest that a pH < 7.25 is linked to a high risk of NIV failure event if a trial might still be considered [7, 8]. The underutilization of NIV in hypercapnic COPD exacerbations suggested by some reports might be at least partially due to these contraindications, which could encourage physicians to immediately proceed with ETI [9].

In Geneva, an emergency mobile unit called “Service Mobile d’Urgence et de Réanimation” (SMUR), staffed by an advanced paramedic and a physician, can be dispatched to assist a regular ambulance in case of life-threatening emergencies [10]. All SMUR vehicles have been equipped by a Hamilton T1 ventilator (Hamilton Medical, Bonaduz, Switzerland) since 2013, and the decision to initiate NIV is made by the physician according to his clinical evaluation. A portable blood gas analyzer (iStat, Abbott Point of Care Inc., Princeton, USA) is available to guide treatment decision and assess the patients’ evolution.

We report here two cases of ARF in COPD patients with severe respiratory acidosis and hypercapnic coma successfully treated by NIV in the prehospital setting.

## Case presentation

Case 1: A 71-year-old woman known for Global Initiative for Chronic Obstructive Lung Disease (GOLD) stage 3 COPD under long-term supplemental oxygen therapy was found in respiratory distress the morning after having received Oxazepam 30 mg for anxiety and insomnia during the previous evening. Upon arrival, prehospital providers noted a Glasgow Coma Scale (GCS) of 6 with no focal deficit, shallow breathing with a respiratory rate over 40 breaths per minute, and a pulsed oxygen saturation of 80% under supplementary oxygen (6 L/min) through nasal cannula. There was no hemodynamic compromise, and the patient was apyretic. Reservoir oxygen face mask was applied and arterial blood gas obtained, showing severe respiratory acidosis (pH 7.17, pCO_2_ 15.6 kPa, pO_2_ 24.1 kPa, bicarbonates 43 mmol/L).

A NIV trial was started using bi-level positive airway pressure ventilation mode, targeting a tidal volume of 6 ml/kg ideal body weight. Patient was under continuous medical surveillance during transport to the emergency room (ER) and showed progressive neurological improvement with a GCS rising to 10. After 60 min of ventilation, a new arterial blood gas sample was drawn showing improvement of her respiratory acidosis (FiO_2_ 21%: pH 7.31, pCO_2_ 10.0 kPa, pO_2_ 7.1 kPa, bicarbonates 37 mmol/L). She was then admitted to ICU to continue NIV, which was successfully continued. ETI was never required, and the patient was transferred to the internal medicine ward after 2 days before being discharged from the hospital after 14 days.

Case 2: A 70-year-old man known for GOLD stage 4 COPD under long-term supplemental oxygen therapy who had been treated by bronchodilators, antibiotics, and steroids for a week, called the ambulance dispatch central because of increasing dyspnea and chest pain. When prehospital providers arrived on site, the patient was unconscious (GCS 3) and in severe respiratory distress with central cyanosis, rapid/shallow breathing. The pulsed oxygen saturation was of 70% under supplementary oxygen (6 L/min) through nasal cannula. He was hemodynamically stable. Ventilation using a bag-valve mask was quickly initiated, and arterial blood gas obtained, showing severe respiratory acidosis (pH 7.07, pCO_2_ > 17.0 kPa, pO_2_ 40.1 kPa, bicarbonates not recorded due to equipment error). The patient had signed a legal document asking not to be resuscitated nor intubated. A bi-level positive airway pressure ventilation NIV trial was started, and both intravenous aspirin and unfractionated heparin were administered. Constant medical surveillance was provided during transport. Upon arrival in the ER after 30 min of NIV, the patient had a GCS of 14. After 70 min of ventilation, a new arterial blood gas sample was drawn showing improvement of his respiratory acidosis (FiO_2_ 32%: pH 7.24, pCO_2_ 12.2 kPa, pO_2_ 7.6 kPa, bicarbonates 38 mmol/L). Pulmonary embolism was ruled out by CT scan and, because of chest pain and elevated cardiac biomarkers, coronary catheterization was performed showing chronic proximal anterior-interventricular artery occlusion that was treated by stenting. The patient was then admitted to the ICU to continue NIV before being transferred to the internal medicine ward after 2 days and finally discharged from the hospital after 20 days.

## Discussion

Though ETI should have been performed in both these patients according to some guidelines [4, 7], use of prehospital NIV allowed us to avoid this high-risk procedure and its potential complications.

The decision to perform ETI in advanced COPD patients must be carefully weighed as such patients frequently present a prolonged and difficult ventilation weaning and as ETI has been linked with an increased mortality rate [11]. Some authors therefore suggest that it might be worth trying NIV even in patients showing altered level of consciousness [5, 6].

In-hospital underutilization of NIV is nevertheless often reported, with ETI being chosen in as many as 60% of patients meeting criteria for NIV trial. The contraindications found in some guidelines are probably among the most important factors leading the physicians to favor immediate ETI [9]. In the prehospital setting, the proportion of NIV underutilization might be even greater, as logistic-related issues might cause delays in both time before first medical contact and NIV instauration. Such delays might lead to further respiratory and neurological compromise, finally making emergent ETI unavoidable.

The cases we reported are examples of situations in which the choice between NIV trial and ETI is particularly challenging. The first patient presented with ARF induced by benzodiazepine intoxication, while the second patient had a COPD exacerbation probably precipitated by NSTEMI, assuming that cardiac ischemia was not a consequence of severe hypoxia. In both cases, we believe that clinical improvement was entirely due to NIV as it was the only effective treatment administered by the prehospital team. Indeed, no flumazenil was given to the first patient despite probable benzodiazepine intoxication, and the second patient was given aspirin and heparin, but no nitrates, diuretics, or inotropic medication.

Even if these two patients presented with ARF of different etiologies, their clinical picture at first medical contact was similar. They both had baseline advanced COPD, a pH < 7.25, and a GCS < 8. Within the first hour, respiratory acidosis regressed (with a pH increase of 0.14 and 0.17 respectively) and neurological status improved (with GCS improving from 6 to 10 and from 3 to 14 respectively) in both cases, making it possible to avoid ETI. For the first patient, NIV was tried because of the potential harmful effects of ETI and mechanical ventilation in the context of advanced COPD. Conversely, while the second patient undoubtedly met criteria for immediate ETI, the procedure was not performed to respect his will. Nevertheless, clinical improvement was even more spectacular in this case, with the patient recovering an almost normal level of consciousness in only 30 min.

The main limitation of this report is that it only describes two cases. Nevertheless, both cases show that NIV could reasonably be attempted in patients in whom ETI should have been performed according to some guidelines. Indeed, though some authors argue that all patients at high aspiration risk should be intubated, it is our opinion that the risks linked to NIV should be weighed against the risks linked to ETI, and that the kind of clinical surveillance that can be provided should be taken into account.

As a physician-staffed emergency mobile unit assists regular ambulances in case of life-threatening emergencies such as ARF in our prehospital system, critical patients are constantly monitored by an emergency physician throughout transport [10]. In a system allowing well trained healthcare providers to immediately react by withdrawing the NIV mask, tilting the patient on the side, suctioning the airways, or even performing immediate rapid-sequence intubation, we believe that NIV can be considered even in situations where ETI is still often considered as a first choice.

In conclusion, for COPD patients presenting ARF, NIV trial could be considered even when relative contraindications such as an altered level of consciousness or a severe respiratory acidosis are present. In such cases, continuous medical surveillance is mandatory, and physicians should be ready to perform ETI should complications arise or the trial fail.

## Data Availability

The datasets used and/or analyzed during the current study are available from the corresponding author on reasonable request.

## Abbreviations

ARF: Acute respiratory failure
COPD: Chronic obstructive pulmonary disease
NIV: Non-invasive ventilation
ETI: endotracheal intubation
ICU: Intensive care unit
GCS: Glasgow Coma Scale
GOLD: Global Initiative for Chronic Obstructive Lung Disease
ER: Emergency room
